# Role of Autophagy in *Ehrlichia*-Induced Liver Injury

**DOI:** 10.3390/cells12091334

**Published:** 2023-05-07

**Authors:** Aditya Kumar Sharma, Nahed Ismail

**Affiliations:** Department of Pathology, College of Medicine, University of Illinois at Chicago, Chicago, IL 60612, USA; aditya06@uic.edu

Autophagy is a cellular process that involves the cell breakdown and recycling of cellular components, such as old, damaged, or abnormal proteins, for important cellular functions including development, immune function, stress, and starvation [1]. Autophagy involves the formation of double-membrane vesicles called autophagosomes that fuse with lysosomes, which contain enzymes that degrade the content of autophagosomes [1,2]. The autophagy mechanism involves a series of steps: initiation, nucleation, elongation, and fusion to lysosomes [3]. The initiation step, or the beginning of autophagy, involves the ULK1 complex dissociating from mTORC1, which causes the formation of an isolated membrane, also known as a phagophore. Next, the nucleation step occurs with the help of the VPS34 protein and beclin complex, which results in the recruitment of proteins for phagophore expansion. Decreased levels of the VPS34–beclin complex in certain tissues upon aging, such as brain tissue, is associated with defective autophagy [3]. After nucleation, phagophore elongation occurs in which the ATG5-ATG12 and LC3-PE conjugation systems play an essential part [4]. This elongation step seals the encompassing cargo and results in the formation of autophagosomes. Finally, the autophagosomes fuse with lysosomes to form autophagolysosomes. In this fusion step, the fusion is regulated by the RAB7 effector protein, which utilizes the HOPS complex and LC3/GABARAP protein [5]. Additionally, the SNARES protein, which has a role in vesicular transport, plays a facilitative role in fusion [6]. Several proteins govern the proper functioning of autophagy, and any aberration in the expression or activation of these proteins can result in impaired autophagic activity. The dysregulation of autophagy has been associated with the pathophysiology of numerous diseases, including cancer, neurological disorders, and metabolic disorders [7].

Human monocytic ehrlichiosis (HME) is a tick-borne infectious disease caused by *Ehrlichia*, which is a Gram-negative obligate intracellular bacterium. *Ehrlichia* targets the liver, which is the main initial site of infection and pathology. Liver dysfunction followed by liver failure, sepsis, and multi-organ failure is the main cause of death in HME [8]. HME presents with non-specific symptoms and often a lack of a history of tick exposure; thus, it is often undiagnosed or misdiagnosed, which is compounded by a lack of highly sensitive and specific diagnostic tests, resulting in delayed treatment [8]. Early treatment with doxycycline can be effective. However, late treatment is frequently ineffective in preventing the disease’s progression to life-threatening sepsis or toxic shock syndrome, which is associated with severe liver damage and multi-organ failure [8]. Interestingly, extensive tissue damage (mainly in the liver) in HME patients is associated with few pathogens in blood and other tissues, suggesting that severity is not due to overwhelming infection, but rather to immunopathology. *Ehrlichia chaffeensis*, the major cause of HME, as well as other *Ehrlichia* species (e.g., *E. canis; Ixodes Ovatus Ehrlichia* (IOE), also known as *E. japonica*; *E.muris* (EM); and *E. muris*-like agent (EMLA)) can cause mild or severe disease depending on the strain virulence and host immune status [8]. Macrophages are the main target cells for *Ehrlichia* species, although *Ehrlichia* species can also infect hepatocytes and endothelial cells in humans [8]. Pathological and clinical findings of mild and fatal HME have been modeled in immune-competent mice using mildly virulent *E. muris* or highly virulent IOE, both of which are closely related to each other and to *E. chaffeensis* [8]. Employing murine models of ehrlichiosis, we have shown that *Ehrlichia*-induced hepatic injury is due to an over-activation of inflammasomes and the type I interferon response causing the induction and expansion of pathogenic natural killer cells, neutrophils, and CD8^+^ T cells, which together cause tissue damage as well as systemic cytokine and chemokine storms [8]. *Ehrlichia*-induced inflammasome activation in macrophages was due to the blocking of autophagy induction and flux that resulted in the accumulation of host and microbial ligands that trigger activations of inflammasomes [8,9]. This editorial focuses on the role of autophagy in the liver in the context of infections, and mainly *Ehrlichia* infection. Here, we will discuss the role of autophagy dysregulation in the pathogenesis of *Ehrlichia*-induced acute liver damage and/or failure. We will also discuss potential therapeutic approaches for autophagy modulation in the treatment of *Ehrlichia*-induced liver injury.

Autophagy is an important mechanism for maintaining cellular homeostasis. Many intracellular bacteria modulate autophagy for intracellular existence and multiplication [10]. Typically, mycobacteria survive inside the macrophage by restricting phagosome maturation [11]. Interestingly, there is growing evidence that autophagy arrests the mycobacterial growth and promotes its degradation inside the cell [12], whereas mycobacteria strategically attempt to block autophagy for their survival. It has been reported that ESAT6, a type VII secretor effector protein, can block autophagic flux [13]. Additionally, SapM, an *M.tb*-secreted acid phosphatase, prevents autophagosome–lysosome fusion via targeting the RAB7 protein, which is involved in endocytosis [14,15]. Recent studies have indicated that autophagy promotes the survival and replication of *Ehrlichia* within macrophages [16]. *Ehrlichia* also modulates autophagy to obtain nutrients for its survival. *E. chaffeensis* hijacks RAB5 to form an RAB5-associated phagocytic complex, which functions as an intracellular transport carrier to transport nutrients from degraded cellular debris to *Ehrlichia* residing within phagosomes [8,16]. Rab5 is a regulator of endocytosis. It controls membrane receptor trafficking and internalization and is a key player in endosome transport [17]. We have recently shown that the blocking of autophagy induction and flux in murine primary macrophages infected with IOE, a virulent species of *Ehrlichia* that cause fatal liver injury and sepsis in mice similar to what has been shown in humans, is due to the TLR9/MYD88-mediated activation of a mammalian target of rapamycin complex 1 (mTORC1), a negative regulator of autophagy [8,18]. Since autophagy is an immune evasion mechanism that is essential for the survival of *Ehrlichia* in macrophages, these data suggest that the MYD88-mediated inhibition of autophagy is a host-protective mechanism. In addition, nutrient availability can also play a role in the regulation of autophagy during ehrlichiosis. *Ehrlichia* infection can induce changes in cellular metabolism, including changes in glucose and fatty acid metabolism, which can impact the availability of nutrients for cellular processes. Studies have shown that nutrient starvation has an impact on mTORC1 and AMPK, which are key regulators of cellular metabolism [19]. In conditions where nutrient and energy levels are low, AMPK activates ULK1, an autophagy initiator, to promote autophagy. In contrast, when nutrients are in abundance, mTORC1 is activated, which limits ULK1 activation [19]. The control of AMPK and mTORC1 is intricate and interconnected. It has been suggested that many AMPK substrates directly or indirectly regulate mTORC1 activity. Studies have shown that AMPK phosphorylates the TSC2 tumor suppressor and essential mTORC1 binding component raptor to reduce mTORC1 activity, which stimulates protein synthesis and cell growth while suppressing autophagy [19]. Furthermore, the immunological response to *Ehrlichia* infection may influence how autophagy is regulated. Anti-inflammatory and immunosuppressive cytokines such as interleukin-10 (IL-10) can inhibit autophagy via the activation of the mTOR-STAT3 pathway [20].

Finally, it needs to be understood that canonical autophagy is a crucial cellular process involved in the defense against intracellular bacteria, and the pathophysiology of liver injury depends significantly on autophagy. Non-canonical autophagy, an alternate autophagy pathway, has been recently described. Non-canonical autophagy involves mechanisms that are beclin-independent mechanisms and do not involve AMPK-mTORC-ULK1 proteins in the initiation of autophagy [21]. These non-canonical mechanisms, such as xerophagy, only require a small subset of canonical mechanism proteins and bypass many steps. Selective autophagy, or xerophagy, gives cells an additional chance to eradicate pathogens that evade conventional autophagy [21]. In *Ehrlichia*, xerophagy and other non-canonical mechanisms have not been investigated. However, it is currently believed that conventional autophagy pathways, which involve proteins such as LC3II and beclin, play a significant role [8].

Furthermore, it is important to note that the regulation of autophagy is cell and tissue specific [8,9]. We recently reported on how the infection of hepatocytes with virulent *Ehrlichia* promotes autophagy in an mTORC1-independent manner, while the same bacteria inhibit autophagy in hepatocytes via mTORC1 activation [8,9]. Further, our studies suggest that the autophagy process in the liver during fatal *Ehrlichia* infection is regulated by type I interferon (IFN-I) receptor (IFNAR) signaling [8,9]. IFNAR signaling in hepatocytes increases bacterial growth and survival by triggering autophagy [8,9]. Future studies are needed to further understand how *Ehrlichia* regulates autophagy in various cell types. Based on these data, we believe that autophagy-based therapeutics approaches can be promising approaches to prevent or treat liver injury in ehrlichiosis. In this Special Issue, we call for further investigations into the role of autophagy in the liver during infectious diseases caused by intracellular pathogens, as well as the advancement of effective autophagy-based therapeutics for the prevention of these diseases.
